# Latent profile analysis of security among patients with COVID-19 infection in mobile cabin hospitals and its relationship with psychological capital

**DOI:** 10.3389/fpubh.2022.993831

**Published:** 2022-11-16

**Authors:** Chao Wu, Jia-ran Yan, Chun-yan He, Jing Wu, Yin-juan Zhang, Juan Du, Ya-wei Lin, Yu-hai Zhang, Chun-ni Heng, Hong-juan Lang

**Affiliations:** ^1^Department of Nursing, Fourth Military Medical University, Shaanxi, China; ^2^Department of Health Statistics, Fourth Military Medical University, Shaanxi, China; ^3^Department of Endocrinology, Tangdu Hospital, Fourth Military Medical University, Shaanxi, China

**Keywords:** latent profile analysis, mobile cabin hospital, patients with COVID-19 infection, security, psychological capital

## Abstract

**Aim:**

COVID-19 patients' security is related to their mental health. However, the classification of this group's sense of security is still unclear. The aim of our research is to clarify the subtypes of security of patients infected with COVID-19, explore the factors affecting profile membership, and examine the relationship between security and psychological capital for the purpose of providing a reference for improving patients' sense of security and mental health.

**Methods:**

A total of 650 COVID-19 patients in a mobile cabin hospital were selected for a cross-sectional survey from April to May 2022. They completed online self-report questionnaires that included a demographic questionnaire, security scale, and psychological capital scale. Data analysis included latent profile analysis, variance analysis, the Chi-square test, multiple comparisons, multivariate logistical regression, and hierarchical regression analysis.

**Results:**

Three latent profiles were identified—low security (Class 1), moderate security (Class 2), and high security (Class 3)—accounting for 12.00, 49.51, and 38.49% of the total surveyed patients, respectively. In terms of the score of security and its two dimensions, Class 3 was higher than Class 2, and Class 2 was higher than Class 1 (all P < 0.001). Patients with difficulty falling asleep, sleep quality as usual, and lower tenacity were more likely to be grouped into Class 1 rather than Class 3; Patients from families with a per capita monthly household income <3,000 and lower self-efficacy and hope were more likely to be grouped into Classes 1 and 2 than into Class 3. Psychological capital was an important predictor of security, which could independently explain 18.70% of the variation in the patients' security.

**Conclusions:**

Security has different classification features among patients with COVID-19 infection in mobile cabin hospitals. The security of over half of the patients surveyed is at the lower or middle level, and psychological capital is an important predictor of the patients' security. Medical staff should actively pay attention to patients with low security and help them to improve their security level and psychological capital.

## Introduction

At present, the COVID-19 pandemic continues to spread among countries and regions, and there is no specific antiviral therapy so far (1, 2). It has greatly changed the global political and economic development model, the trend of international relations, and even people's daily lives (3). The scope, scale, destructive power, and consequences of COVID-19 are unprecedented in history (4, 5). It has a strong transmission capacity, and once human beings are infected with the novel coronavirus, they can develop chest pain, cough, expectoration, fever, and other infection symptoms (6, 7). Severe infections can lead to respiratory failure or even multiple organ failure (8). To some extent, COVID-19 has created panic and fear among people.

Now in China, the COVID-19 pandemic is still existing, and the fight against the pandemic is ongoing. In March 2022, COVID-19 broke out on a large scale in Shanghai, China. By the end of May, there had been more than 600,000 confirmed cases of COVID-19 and asymptomatic infections in Shanghai. As an effective epidemic control measure for COVID-19 pandemic, the mobile cabin hospital is an effective way to cut off the potential route of infection in society (9). Medical staff are dispatched to mobile cabin hospitals to take care of patients who test positive for the virus but show no severe symptoms. This can relieve the pressure of hospital reception and achieve the goal of zero transmission in communities outside quarantine (10, 11). The mobile cabin hospital has many advantages, such as its rapid deployment, good mobility, and strong environmental adaptability, and can apply well to emergency medical rescue tasks, so it has been highly valued by various countries (12, 13). In this round of the pandemic in Shanghai, a great number of cabin hospitals were needed to curb the COVID-19 pandemic, and exhibition halls, gymnasiums, and other buildings were transformed into mobile cabin hospitals.

As a kind of stressor, the outbreak of an epidemic can easily lead to individual psychological stress (14, 15). Research shows that the psychological status of patients with diagnosed COVID-19 is not optimistic, and they have varying degrees of anxiety and panic due to their worrying about the prognosis of the disease (16, 17). Patients who entered mobile cabin hospitals for centralized isolation treatment were highly concentrated in a relatively narrow but open space. Such an unfamiliar environment can easily induce mental health problems and hinder the rehabilitation of the disease (18). Furthermore, with the virus becoming less and less aggressive, most patients will have mild or moderate symptoms, to whom more attention should be paid. The source of their negative emotions and mental health problems is mainly their uncertainty and lack of security (19, 20).

Sense of security refers to the individual's feeling of confidence and freedom when faced with fear and danger (21), as well as the psychological feeling when dealing with these risks (22). It is one of the important factors affecting mental health and is mainly manifested as a sense of certainty and control (23). In recent years, because security is a basic psychological need, it has received a lot of attention and been widely studied. When individuals' security is threatened, they will be nervous and afraid, and their normal life and work will even be affected (24). Since COVID-19 pandemic is a public health emergency and is highly contagious, it threatens people's security (25). When the confirmed cases enter the mobile cabin hospital for centralized isolation, facing the unfamiliar environment and uncertain conditions, their security is threatened. This will cause them to have negative emotions such as anxiety and fear and affect the treatment of the disease (26). Therefore, patients' security in mobile cabin hospitals needs attention.

Psychological capital refers to a positive psychological state that individuals show in the process of growth and development, including self-efficacy, hope, optimism, and tenacity (27). Research shows that psychological capital is positively correlated with a sense of security; that is, individuals with good psychological capital will have a strong sense of security (28). In the face of threats, psychological capital has a good buffer effect to counter fear and anxiety (29). Under the same situation, the security of individuals with high psychological capital is higher than that of those with low psychological capital (30, 31).

However, currently there are few investigations into the security of patients with COVID-19 infection in mobile cabin hospitals, and their security levels are not clear. Moreover, there are rare researches on the relationship between the security and psychological capital of patients with COVID-19 infection in mobile cabin hospitals. Therefore, the purpose of our study is to take the COVID-19 patients in mobile cabin hospitals during the outbreak of COVID-19 in Shanghai as the research objects and to investigate their sense of security and related influencing factors to provide a reference for further optimizing the management of mobile cabin hospitals and improving the patients' sense of security.

## Methods

### Sample size

The sample size was equal to 10 times the number of items being tested. There were 49 items in our questionnaire. Therefore, the calculation formula of sample size was *n* = (7 items + 16 items + 26 items) × 10 = 490, which meant that at least 490 subjects were required for this study. Considering an expected sample loss rate of 20%, the sample size needed be further expanded. Therefore, the final sample size required was *n* = 490 ÷ (1–20%) ≈612.5, and the final sample size required was 613 at least.

### Participants

The study was conducted in accordance with the Declaration of Helsinki (32) and the guidelines of the Air Force Medical University. Participants were patients with confirmed COVID-19 recruited from a mobile cabin hospital during the outbreak of COVID-19 in Shanghai. All patients were informed that participation in this study was voluntary. They could withdraw from the study at any time for any reason, and the questionnaires were completed anonymously. The inclusion criteria were COVID-19 patients in a mobile cabin hospital. The exclusion criteria were those who had recently experienced major life events and those who were unwilling to participate in the survey. A total of 650 patients with COVID-19 were selected for the cross-sectional survey from April to May 2022. However, 18 subjects withdrew from the survey, 13 did not fill out the questionnaire completely, and 11 filled out the questionnaire with too much homogeneity, which was regarded as an invalid questionnaire. The final sample included 608 patients (270 men, 338 women) from the mobile cabin hospital, aged 11–76 years (*M*_*age*_ = 38.08, *SD* = 13.47).

### Measures

#### Descriptive measures

Data on demographic variables (i.e., sex, age, place of residence, education background, marital status, monthly income per capita in family, sleep quality after diagnosis with COVID-19) were collected through a self-reported questionnaire.

#### Security

Participants' security levels were measured using the Security Scale (33). The Security Scale is applicable to Chinese cultural background and is widely used in China, as well as has good reliability and validity among Chinese people (34, 35). The Security Scale contains 16 items and two dimensions: interpersonal security and certainty in control. Among them, the interpersonal security dimension contains eight items, which mainly reflect the individual's security in the process of interpersonal communication. The certainty in control dimension contains eight items, which mainly reflect the individual's prediction of life and their sense of certainty and control. The scale adopts Likert's 5-level scoring method, with 1–5 points from very consistent to very inconsistent, respectively. The highest possible score is 80, with higher scores indicating more security. For example, “I always worry that something bad will happen.” The Cronbach's alpha for the interpersonal security dimension and the certainty in control dimension were 0.868 and 0.934, and for the total scale was 0.902.

#### Psychological capital

Participants' psychological capital was measured using the Psychological Capital Scale (36). The psychological capital Scale is widely used in China and has good reliability and validity among Chinese people (37, 38). The scale contains 26 items and four dimensions: self-efficacy, hope, optimism, and tenacity. The higher the score of the scale, the higher the positive tendency of psychological capital. The scale adopts Likert's 7-level scoring method, with 1–7 points from completely non-compliant to fully compliant, respectively. The Cronbach's alpha coefficient of the scale was 0.873 and ranged between 0.855 and 0.920 for the four dimensions.

### Procedure and data analysis

Before beginning the formal investigation, we trained the researchers to ensure the quality of the research. With the consent of the mobile cabin hospital managers, researchers explained the purpose of the survey to the patients to obtain their approval and support prior to data collection, and the patients gave their consent to participate in the research. With the help of head nurses in the mobile cabin hospital, questionnaires were distributed to the patients. The questionnaire was collected on the spot. We eliminated invalid questionnaires that were not filled in completely and answered randomly. The method of double check was used to input the data of the valid questionnaires to ensure accuracy.

SPSS 26.0 statistical software and Mplus 8.3 were used for statistical analysis. Descriptive statistics (mean, standard deviation, frequency and percentage) were used to describe the sample's characteristics. The Chi-square test and variance analysis were used to screen statistically significant indicators. Logistic regression analysis was used to evaluate the influencing factors of potential categories. The data for security were entered into the latent profile analysis, with one class initially and additional classes added incrementally until a unique solution could not be determined with maximum likelihood methods. The latent profile analysis model evaluation indicators included the Akaike information criterion (AIC), Bayesian information criterion (BIC), sample-size-adjusted BIC (aBIC), bootstrapped likelihood ratio test (BLRT), Lo-Mendell-Rubin (LMR) adjusted likelihood ratio test, Vuong-Lo-Mendell-Rubin likelihood ratio test (VLMR), and entropy. A higher entropy value, which is an important indicator, indicates a more accurate classification of the model. The smaller the AIC, BIC, and aBIC values, the better the model fit. LMR, BLRT, and VLMR are often used in model comparison, and a significant *P*-value indicates that K model categories are better than K – 1 model categories. The best-fitting models were selected through comprehensive evaluation of the above indexes, and the security among patients infected with COVID-19 in the mobile cabin hospital was divided into different categories. Hierarchical regression analysis was used to explore the impact of psychological capital on the security of patients. All tests conducted were two-sided, with a significance level of 0.05.

### Ethical considerations

Our research was in accordance with the ethical standards formulated in the Declaration of Helsinki (39) and was confirmed by the Fourth Military Medical University ethics committee approval (KY20224143-1). Informed consent was provided by the participants prior to their participation. The survey was anonymous, and the confidentiality of the information was assured.

## Results

### Common method deviation

The Harman single-factor method was used to test the common method deviation. The variance explained by the maximum factor variance was 27.8%, less than the critical value of 40% (40). The statistical test results showed that there was no significant common method bias in the measurement.

### Exploratory latent profile analysis

**Figure 1:**
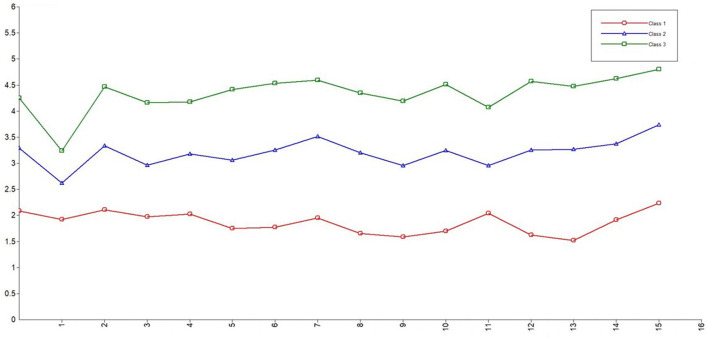
The latent profiles of the security of patients infected with COVID-19.

The best-fitting latent profile analysis was the three-class model (Table 1). The *P*-values of the LMR test (<0.001), VLMR test (<0.001), and BLRT test (<0.001) of the three-class model were the smallest, suggesting that this model was statistically significant at the α = 0.05 level. This model had the highest entropy value (0.920) and the lower AIC (28,730.713), BIC (29,021.784), and aBIC (28,812.250). Figure 1 shows the subtypes of patients' security (Classes 1, 2, and 3); the proportion of each type was 12.00, 49.51, and 38.49%. According to this model, they could be distinguished as having relatively low (Class 1), medium (Class 2), or high (Class 3) security. The correct classification probabilities mean the average latent class probabilities for the most likely latent class membership by latent class. The larger the proportion, the more accurate the result. In order to further verify the reliability of the classification results, the correct classification probabilities of the three categories of patients' safety were calculated to be 97.1, 96.6, and 95.7% respectively (Table 2).

**Table 1:** Model fit indexes of latent profile analysis (*N* = 608).

**Model**	**K**	**AIC**	**BIC**	**aBIC**	**Entropy**	**LMR**	**VLMR**	**BLRT**	**Category probability**
One-profile	32	32,574.753	32,715.878	32,614.286	–	–	–	–	–
Two-profile	49	29,853.625	30,069.723	29,914.159	0.907	<0.001	<0.001	<0.001	49.67/50.33
**Three-profile**	**66**	**28,730.713**	**29,021.784**	**28,812.250**	**0.920**	**<0.001**	**<0.001**	**<0.001**	**12.00/49.51/38.49**
Four-profile	83	28,401.722	28,767.767	28,504.261	0.888	0.002	0.002	<0.001	10.86/31.91/39.97/17.27
Five-profile	100	28,206.084	28,647.102	28,329.625	0.909	0.453	0.449	<0.001	38.65/11.02/31.74/1.65/16.94

AIC, Akaike information criterion; BIC, Bayesian information criterion; ABIC, same-size adjusted Bayesian information criterion; LMR, Lo-Mendell-Rubin likelihood ratio test; VLMR, Vuong-Lo-Mendell-Rubin likelihood ratio test; BLRT, Bootstrapped likelihood ratio test.

**Table 2:** Average latent class probabilities for most likely latent class membership (row) by latent class (column).

**Class**	**C1 (%)**	**C2 (%)**	**C3 (%)**
C1	**0.971**	0.029	0.000
C2	0.005	**0.966**	0.029
C3	0.000	0.043	**0.957**

C1: Class 1, C2: Class2; C3: Class 3.

### Security of patients with COVID-19 in different categories and characteristics of the different classes

Table 3 presents the security and its two dimensions of the three classes. The results of the analysis of variance of the total score and two dimensions of patient security in each group were statistically significant (*F* = 1,329.070, *P* < 0.001; *F* = 645.427, *P* < 0.001; *F* = 1,141.546, *P* < 0.001). Further least significant difference (LSD) analysis showed that Class 1 < Class 2 < Class 3 in terms of security and its two dimensions (*P* < 0.001). Univariate analysis showed that there were significant differences among the three groups in educational level (χ^2^ = 9.956, *P* = 0.007), family income (χ^2^ = 22.936, *P* = 0.001), psychological capital (*F* = 35.907, *P* < 0.001), and its four dimensions (*F* = 52.296, *P* < 0.001; *F* = 43.750, *P* < 0.001; *F* = 23.610, *P* < 0.001; *F* = 7.192, *P* = 0.001). There was no difference among the three groups in other demographic characteristics (Table 4).

**Table 3:** Security of patients with COVID-19 in different categories.

	***N***	**Security**	**Interpersonal security**	**Certainty in control**
C1: Low security	73	29.95 ± 7.79	15.68 ± 4.77	14.26 ± 4.28
C2: Moderate security	301	51.12 ± 5.23	25.13 ± 3.91	25.99 ± 3.20
C3: High security	234	69.46 ± 6.54	33.87 ± 4.03	35.59 ± 3.67
*F*		1,329.070	645.427	1,141.546
*P*		<0.001	<0.001	<0.001
LSD		C1 < C2 < C3	C1 < C2 < C3	C1 < C2 < C3

**Table 4:** The differences in the security types of COVID-19 patients in demography and psychological capital.

**Variable**	**Respondents**	**Low security**	**Moderate security**	**High security**	**χ^2^/*F***	***P***
**Gender**						
Male	270 (44.41%)	29 (39.73%)	136 (45.18%)	105 (44.87%)	0.742	0.690
Female	338 (55.59%)	44 (60.27%)	165 (54.82%)	129 (55.13%)		
**Age**					
<30	202 (33.22%)	23 (31.50%)	107 (35.55%)	72 (30.77%)	4.245	0.374
30–50	269 (44.24%)	31 (42.47%)	123 (40.86%)	115 (49.14%)		
>50	137 (22.53%)	19 (26.03%)	71 (23.59%)	47 (20.09%)		
**Place of residence**						
City	380 (62.50%)	42 (57.53%)	189 (62.79%)	149 (63.68%)	0.917	0.632
Countryside	228 (37.50%)	31 (42.47%)	112 (37.21%)	85 (36.32%)		
**Education background**						
Junior college or below	499 (82.07%)	65 (89.04%)	256 (85.05%)	178 (76.07%)	9.956	0.007
Undergraduate or above	109 (17.93%)	8 (10.96%)	45 (14.95%)	56 (23.93%)		
**Marital status**						
Unmarried	198 (32.57%)	23 (31.51%)	105 (34.88%)	70 (29.91%)	2.589	0.629
Married	382 (62.83%)	47 (64.38%)	180 (59.80%)	155 (66.24%)		
Widowed or separated	28 (4.61%)	3 (4.11%)	16 (5.32%)	9 (3.85%)		
**Monthly income per capita in family (yuan)**						
<3,000	126 (20.72%)	25 (34.25%)	66 (21.93%)	35 (14.96%)	22.936	0.001
3,000–5,000	224 (36.84%)	26 (35.62%)	117 (38.87%)	81 (34.62%)		
5,001–10,000	163 (26.81%)	23 (17.81%)	83 (27.57%)	67 (28.63%)		
>10,000	95 (15.63%)	9 (12.33%)	35 (11.63%)	51 (21.79%)		
**Sleep quality after diagnosis with COVID-19**						
Have trouble falling asleep	80 (13.16%)	15 (20.55%)	39 (12.96%)	26 (11.11%)	6.531	0.366
Poor sleep	165 (27.14%)	17 (23.29%)	87 (28.90%)	61 (26.07%)		
As usual	307 (50.49%)	37 (50.68%)	148 (49.17%)	122 (52.14%)		
Good sleep	56(9.21%)	4 (5.48%)	27 (8.97%)	25 (10.68%)		
Psychological capital	110.39 ± 22.70	97.36 ± 34.30	106.90 ± 19.49	118.94 ± 18.65	35.907	<0.001
Self-efficacy	28.47 ± 6.50	24.16 ± 9.34	27.25 ± 5.73	31.38 ± 4.94	52.296	<0.001
Hope	27.91 ± 6.75	23.59 ± 9.30	26.82 ± 6.10	30.66 ± 5.38	43.750	<0.001
Tenacity	27.17 ± 6.17	24.22 ± 8.99	26.40 ± 5.46	29.09 ± 5.35	23.610	<0.001
Optimistic	26.83 ± 5.54	25.38 ± 8.33	26.42 ± 4.90	27.82 ± 5.08	7.192	0.001

### Influencing factors of security in different groups of patients with COVID-19

The security of patients with COVID-19 determined by latent profile analysis was used as the dependent variable, and Class 3 was used as the control group. Compared with Class 3, those with trouble falling asleep (OR = 6.572, *P* < 0.05), sleep quality as usual (OR = 3.693, *P* < 0.05), and lower tenacity (OR = 0.831, *P* < 0.05) were more likely to be grouped into Class 1. Those with a monthly per capita family income <3,000 (OR_class1_ = 3.131, *P* < 0.05; OR_class2_ = 2.383, *P* < 0.05), lower self-efficacy (OR_class1_ =0.678, *P* < 0.001; OR_class2_ = 0.783, *P* < 0.001), and lower hope (OR_class1_ = 0.739, *P* < 0.001; OR_class2_ = 0.868, *P* < 0.001) were more likely to be grouped into Classes 1 and 2 (Table 5).

**Table 5:** The multifactor analysis of security of patients infected with COVID-19 by logistic regression.

**Variable**	**C1 VS C3**					**C2 VS C3**				
	**β**	**SE**	**OR**	**95%CI**	***P***	**β**	**SE**	**OR**	**95%CI**	***P***
**Gender (take female as reference)**										
Male	0.171	0.324	1.187	(0.630, 2.238)	0.596	0.219	0.203	1.244	(0.836, 1.852)	0.281
**Age (take** **>50 as reference)**										
<30	−0.438	0.529	0.645	(0.229, 1.820)	0.407	−0.291	0.342	0.474	(0.382, 1.462)	0.395
30–50	−0.175	0.404	0.839	(0.380, 1.853)	0.665	−0.185	0.263	0.831	(0.496, 1.393)	0.483
**Place of residence (take countryside as reference)**										
City	0.099	0.342	1.104	(0.564, 2.160)	0.773	0.166	0.222	1.181	(0.764, 1.824)	0.454
**Education background (take undergraduate or above as reference)**										
Junior college or below	0.422	0.494	1.525	(0.579, 4.017)	0.393	0.324	0.273	1.383	(0.809, 2.363)	0.236
**Marital status**										
Unmarried	1.084	0.909	2.958	(0.498, 17.572)	0.233	0.153	0.537	1.166	(0.407, 3.338)	0.775
Married	0.847	0.826	2.332	(0.462, 11.765)	0.305	−0.176	0.483	0.839	(0.325, 2.161)	0.716
**Monthly income per capita in family (yuan) (take** **>10,000 as reference)**										
<3,000	1.142	0.571	3.131	(1.023, 9.589)	**0.046**	0.869	0.380	2.383	(1.131, 5.022)	**0.022**
3,000–5,000	−0.093	0.536	0.911	(0.319, 2.603)	0.862	0.323	0.328	1.382	(0.726, 2.629)	0.324
5,001~10,000	−0.313	0.546	0.731	(0.251, 2.130)	0.566	0.221	0.317	1.247	(0.671, 2.320)	0.486
**Sleep quality after diagnosis with COVID-19 (take good sleep as reference)**										
Have trouble falling asleep	1.883	0.778	6.572	(1.429, 30.219)	**0.016**	0.686	0.468	1.987	(0.794, 4.969)	0.142
Poor sleep	1.142	0.735	3.134	(0.742, 13.238)	0.120	0.602	0.415	1.825	(0.810, 4.115)	0.147
As usual	1.306	0.662	3.693	(1.008, 13.527)	**0.049**	0.431	0.365	1.539	(0.753, 3.144)	0.237
**Psychological capital (take optimistic as reference)**										
Self-efficacy	−0.388	0.072	0.678	(0.589, 0.780)	**<0.001**	−0.244	0.048	0.783	(0.712, 0.861)	**<0.001**
Hope	−0.302	0.070	0.739	(0.644, 0.848)	**<0.001**	−0.141	0.047	0.868	(0.791, 0.953)	**0.003**
Tenacity	−0.185	0.083	0.831	(0.706, 0.978)	**0.026**	−0.090	0.056	0.914	(0.820, 1.019)	0.106

SE: Standard error, OR: Odds ratio, CI: Confidence interval.

### Hierarchical regression analysis of COVID-19 patients' security

Table 6 shows the results of hierarchical regression analysis with security as the dependent variable. The results showed that family income and sleep quality had entered the regression equation of the security of COVID-19 patients (*F* = 3.182, *R*^2^ = 0.089, *P* < 0.001). On the basis of model 1, four dimensions of psychological capital were included in model 2 (*F* = 10.093, *R*^2^ = 0.275, *P* < 0.001). Variance expansion factors were <10, indicating that there was no obvious collinearity among variables. Psychological capital was an important predictor of patients' security that could independently explain 18.70% of its variation (Δ*R*^2^ = 0.187, *P* < 0.001).

**Table 6:** Hierarchical regression analysis of psychological capital on the security of COVID-19 patients.

**Variable**	**M1**					**M2**				
	***b***	***Sb***	***b'***	***t***	***P***	***b***	***Sb***	***b'***	***t***	***P***
Constant	47.555	4.448		10.692	<0.001	26.777	4.778		5.605	<0.001
**Family income (yuan per month)**										
3,000–5,000	4.159	1.583	0.142	2.627	0.009	4.643	1.423	0.158	3.263	0.001
5,001–10,000	7.459	1.840	0.234	4.053	<0.001	6.190	1.652	0.194	3.747	<0.001
>10,000	9.274	2.186	0.238	4.242	<0.001	6.672	1.970	0.171	3.387	0.001
**Sleep quality after diagnosis with COVID-19**										
Good sleep	7.324	2.476	0.150	2.958	0.003	6.732	2.232	0.138	3.015	0.003
**Psychological capital**										
Self-efficacy						0.796	0.154	0.365	5.182	<0.001
Hope						0.477	0.159	0.227	2.993	0.003
Tenacity						0.125	0.168	0.054	0.744	0.457
Optimistic						−0.685	0.150	−0.269	−4.570	<0.001
*F*			3.182^***^					10.093^***^		
*R* ^2^			0.089					0.275		
Adjusted *R*^2^			0.061					0.248		
Δ*R*^2^								0.187^***^		

M1, Model 1; M2, Model 2.

*** : P < 0.001.

## Discussion

### Necessity of the study on the security of patients with COVID-19 infection in mobile cabin hospitals

The establishment of mobile cabin hospitals to treat a large number of patients with mild or moderate COVID-19 is an effective way to deal with the outbreak of the pandemic and block its spread at the social level (41). In the face of the special isolation environment in these hospitals, patients' security is threatened, which has an impact on their psychological health and disease treatment. However, there is a lack of research on the sense of security of this group, and limited studies have examined whether psychological capital might impact the sense of security of patients with COVID-19 infection in mobile cabin hospitals. Therefore, the purpose of this study was to clarify the subtypes of security of patients infected with COVID-19, to explore the influencing factors of different types of security, and to examine the relationship between patients' security and psychological capital. To our knowledge, this is the first research to study the security of patients with COVID-19 infection in mobile cabin hospitals.

### More attention should be paid to patients with low sense of security

Our study used latent profile analysis to classify patients' sense of security into three categories: low security (Class 1), moderate security (Class 2), and high security (Class 3). Among them, Class 2 had the largest number, accounting for 49.51%, which indicates that nearly half of the patients had moderate security. Although the number of patients in Class 1 was the lowest, accounting for 12%, this group needs the most attention.

More and more attentions have been paid to patients' sense of security in recent years, and it is an important aspect of patient-centered care (42). Previous studies have shown that patients with low sense of security were more worried about their economic situation (43). A longitudinal study showed that patients with high security always had good psychological health (44). The patient's sense of security includes many influencing factors, such as external environmental factors and self-related factors, such as economic and psychological conditions (45, 46). In our study, we explored the sense of security of patients with COVID-19 from different perspectives, which were showed in Tables 4, 5. However, the results in Tables 4, 5 were not consistent because that a multivariable logistic regression model was performed to include all independent variables.

Those whose per capita family income was <3,000 tend to have a low or medium sense of security. This is because under COVID-19, people in controlled zones are restricted from going to work, which has a great impact on people's income, especially in low-income families (47, 48). Such people have to worry about disease, but also their living expenses and loan repayment, so they are more likely to have a low sense of security. Furthermore, our results showed that the sleep quality of patients after diagnosed with COVID-19 also affected their security, and that the two are closely related. Our results are consistent with Hoyniak et al.'s (49) finding that emotional security is conducive to sleep. A cross-sectional study showed that COVID-19 is associated with changes in sleep schedule and in the quantity and quality of nighttime sleep because of the new stressors, altered roles, and uncertainties about health and economic security (50). Therefore, in the management and nursing of COVID-19 patients in mobile cabin hospitals, from the perspective of improving their security, we should focus on patients with low income and poor sleep quality.

### The important role of psychological capital in patients' safety

Psychological capital belongs to the category of positive psychology, which emphasizes individual strength and enthusiasm (51, 52). Our study shows that COVID-19 patients with good psychological capital tend to have a high sense of security, and it is an important predictor of patients' security that can independently explain 18.70% of the variation in security, which is consistent with the results of Eweida et al. (28). In the hierarchical regression analysis of this study, tenacity was not significant in model 2. The reason for this may be that tenacity is a persistent quality that has few impacts on the sense of security. Research shows that individuals with low psychological capital have a low sense of security (53, 54). Psychological capital can buffer the uncertainty, stress, and anxiety of patients in mobile cabin hospitals, which can easily lead to an increase in security (55, 56). Therefore, managers of shelter hospitals can improve patients' security by improving their psychological capital level (28). Medical staff can guide patients to adjust their mentality and help them to adjust their negative emotions, which is conducive to the acquisition of a sense of security. According to Bandura's social cognitive theory, people who have subjective initiative can actively adapt to and change the environment (57). Research shows that mindfulness can improve the level of individual psychological capital (58). Therefore, mindfulness-based cognitive therapy can be conducted for patients in shelters to treat and alleviate their emotional and psychological problems, such as anxiety, depression, and impulsiveness, so as to improve their self-efficacy, hope, and optimism (59, 60). Individuals with a high level of psychological capital are more willing to take initiative and face difficulties and turning points, are more optimistic, confident, and hopeful, and can recover quickly even if they encounter setbacks (61).

## Limitations

There are some limitations to our study. Firstly, because of the impact of the pandemic, our research group only conducted a questionnaire survey on COVID-19 patients in the shelter unit within our own management, and we did not investigate the patients in the whole mobile cabin hospital. Secondly, our study was conducted in the form of self-report questionnaires, and the results tended to be subjective. Third, we only preliminarily explored the relationship between the psychological capital and the security of patients, without combining the clusters of security. In future research, we will further explore the relationship between security and psychological capital in combination with the type of security.

## Conclusion

Our study explored the characteristics of security among patients with COVID-19 infection in mobile cabin hospitals and its relationship with psychological capital. Based on latent profile analysis, we identified their security as low security (Class 1), moderate security (Class 2), or high security (Class 3), accounting for 12.00, 49.51, and 38.49%, respectively, of the total number of patients. The predictors of COVID-19 patients' security were monthly income per capita in the family, sleep quality, and psychological capital. Psychological capital was an important predictor of security that could independently explain 18.70% of the variation in the patients' security. All these predictive factors are of great significance to improve COVID-19 patients' security, which managers of shelter hospitals should pay attention to.

## Data availability statement

The raw data supporting the conclusions of this article will be made available by the authors, without undue reservation.

## Author contributions

CW, J-rY, and C-yH wrote the main manuscript text. H-jL, JW, and C-nH distributed questionnaires. Y-jZ and JD contributed to the writing and revision of articles. Y-wL and Y-hZ contributed to the analysis and processing of data. All authors contributed to the article and approved the submitted version.

## Conflict of interest

The authors declare that the research was conducted in the absence of any commercial or financial relationships that could be construed as a potential conflict of interest.

## Publisher's note

All claims expressed in this article are solely those of the authors and do not necessarily represent those of their affiliated organizations, or those of the publisher, the editors and the reviewers. Any product that may be evaluated in this article, or claim that may be made by its manufacturer, is not guaranteed or endorsed by the publisher.
